# Understanding carbon utilization routes between high and low starch-producing cultivars of cassava through Flux Balance Analysis

**DOI:** 10.1038/s41598-019-39920-w

**Published:** 2019-02-27

**Authors:** Porntip Chiewchankaset, Wanatsanan Siriwat, Malinee Suksangpanomrung, Opas Boonseng, Asawin Meechai, Morakot Tanticharoen, Saowalak Kalapanulak, Treenut Saithong

**Affiliations:** 1 Division of Biotechnology, School of Bioresources and Technology, King Mongkut’s University of Technology Thonburi (Bang Khun Thian), Bangkok, 10150 Thailand; 2 Systems Biology and Bioinformatics Research Group, Pilot Plant Development and Training Institute, King Mongkut’s University of Technology Thonburi (Bang Khun Thian), Bangkok, 10150 Thailand; 3 Plant Molecular Genetics and Biotechnology Laboratory, National Center for Genetic Engineering and Biotechnology, Thailand Science Park, Pathumthani, 12120 Thailand; 4 Rayong Field Crops Research Center, Department of Agriculture, Rayong, 21150 Thailand; 5 Department of Chemical Engineering, Faculty of Engineering, King Mongkut’s University of Technology Thonburi (Bang Mod), Bangkok, 10140 Thailand; 6 School of Bioresources and Technology, King Mongkut’s University of Technology Thonburi (Bang Khun Thian), Bangkok, 10150 Thailand; 7 Bioinformatics and Systems Biology Program, School of Bioresources and Technology, King Mongkut’s University of Technology Thonburi (Bang Khun Thian), Bangkok, 10150 Thailand

## Abstract

Analysis of metabolic flux was used for system level assessment of carbon partitioning in Kasetsart 50 (KU50) and Hanatee (HN) cassava cultivars to understand the metabolic routes for their distinct phenotypes. First, the constraint-based metabolic model of cassava storage roots, rMeCBM, was developed based on the carbon assimilation pathway of cassava. Following the subcellular compartmentalization and curation to ensure full network connectivity and reflect the complexity of eukaryotic cells, cultivar specific data on sucrose uptake and biomass synthesis were input, and rMeCBM model was used to simulate storage root growth in KU50 and HN. Results showed that rMeCBM-KU50 and rMeCBM-HN models well imitated the storage root growth. The flux-sum analysis revealed that both cultivars utilized different metabolic precursors to produce energy in plastid. More carbon flux was invested in the syntheses of carbohydrates and amino acids in KU50 than in HN. Also, KU50 utilized less flux for respiration and less energy to synthesize one gram of dry storage root. These results may disclose metabolic potential of KU50 underlying its higher storage root and starch yield over HN. Moreover, sensitivity analysis indicated the robustness of rMeCBM model. The knowledge gained might be useful for identifying engineering targets for cassava yield improvement.

## Introduction

Cassava (*Manihot esculenta* Crantz) is a promising staple crop for attaining food sufficiency in the 21^st^ century^1^ for several reasons. First, cassava is a primary source of calories for more than 800 million people every year especially in Africa^2^, and its roots contain high amount of starch that usually range from 70 to 90% on dry matter basis depending on variety^3^. Next, cassava is a resilient crop and grows in marginal environments, typically deficient in rainfall and soil nutrients, where most food crops cannot survive^4,5^. Besides its utilization as food, cassava starch serves as raw material for a wide range of industries, for example, pharmaceuticals, textiles, plywood, paper, and bioethanol production^1^. Despite its resilience, cassava production is affected by unpredictable weather events that are associated with global climate change^2,6^. In 2015, atypically low level of rainfall together with high temperature resulted in cassava yield loss in Tanzania^7^. Cassava productivity in sub-Saharan Africa declined at an annual rate of 0.024 tons ha^−1^ from 2004 to 2014^8^. To ensure adequate supply of carbohydrate for the rapidly growing global population, estimated to reach 11.2 billion people by year 2100^9^, research is needed to develop climate resilient cultivars with improved productivity, while minimizing resource use (e.g. land, water, and nutrients).

Conventional and molecular plant breeding techniques have enabled the development of cultivars with superior plant phenotypes such as high yields, resistance to diseases, and tolerance to abiotic stress^10^, but both are limited in scope. They typically allow for the improvement of selected traits of interest and do not cover all underlying attributes of the genetic background. For example, Kasetsart 50 (KU50), a Thai cultivar, was bred to enhance yield and starch content^11,12^, while the MH97/296, a Ugandan cultivar, was bred to improve resistance to cassava mosaic disease^13^. Crop phenotype is determined not only by heredity, but also by the interaction between underlying genetics (G) and environment (E), i.e. P = G × E. Srihawong *et al*.^14^ showed that root yield of KU50 and Hanatee (HN) cassava cultivars vary with age of plant and soil moisture. Hence, understanding the combinatorial effect of both factors on phenotype is crucial for cassava trait improvement.

In the post-genomic era, *in silico* metabolic modeling has been adopted as a strategy to investigate the metabolic regulation behind the expressed phenotype under the constraints of genetic background and exposed environment. Flux balance analysis (FBA), a well-established approach for constraint-based modeling, enables the quantitative study of metabolic behavior, i.e. net flux distribution, in the genome-scale metabolic pathway of cells composed of all cellular biochemical reactions according to the genetic information of a given organism. An optimal flux distribution is proposed by optimizing the objective function such as maximum biomass yield or minimum ATP production^15^. Flux balance analysis has been applied to study the metabolic fluxes underlying growth and development of microorganisms, animals, and plants as well as physiological responses to prevailing environment^16–18^. The first plant metabolic model was developed for *Arabidopsis thaliana*, and was employed to synthesize the main biomass components in non-compartmentalized heterotrophic cell suspension culture. First, the active reaction fluxes were minimized and then the model was applied to identify the total ATP demand for cell growth and maintenance^19^. FBA has since been used to study the growth and development of barley seed endosperm^20,21^, oilseed rape embryos^22–24^, rice leaf cells^25^, and tomato fruit^26^, and to investigate the metabolic adaptation of rice to flood and drought stresses^27^. The ultimate aim of these comprehensive studies was to explore its rationale towards sustainable improvement of crop yield. However, there are no metabolic models for starchy root crops to date.

Cassava roots are the largest carbohydrate sink of all starchy staple crops, but there is limited knowledge of the underlying carbon assimilation mechanism; thus, carbon assimilation is of huge research interest. It has been suggested that yield potential of cassava is related to photosynthetic efficiency and carbon partitioning^8,28^. El-Sharkawy and De Tafur^29^ found that photosynthetic efficiency is likely to have a limited effect on cassava yield as similar photosynthetic rate was found in cultivars with quite distinct phenotypes, i.e. different leaf canopy size and root yield. On the other hand, cassava has already high photosynthetic efficiency among C3 plants^30^, and the gap of improvement for this factor may be little. Hence, breeding for photosynthetic efficiency is not only challenging but also may be futile.

This work was therefore conducted to investigate the carbon flux partition in cassava via constraint-based metabolic modeling of carbon assimilation in storage roots of two distinct cassava cultivars, KU50—with high root and starch yield, and HN—with low root and starch yield. We hypothesized that the distinct starch yield in these cultivars is a result of differences in carbon flux partitioning in cellular metabolism of roots that refers to dissimilarity of carbon utilization in root biomass production. KU50 may preferably utilize photosynthates towards starch biosynthesis and other root biomass, whereas HN may rather expend carbon substrates for other cellular activities, such as substrate consumption (e.g. respiration) to support plant growth. The compartmentalized carbon assimilation metabolic model of cassava roots for KU50 and HN was developed from the MeRecon network^31^. It contained 393 metabolites which correspond to 468 reactions. These reactions were scoped to cover the primary metabolism involved in conversion of sucrose to biomass compounds of cassava roots. The model was compartmentalized into cytosol, plastid, and mitochondria to imitate subcellular metabolism in cassava root cells. The constraint-based metabolic model of cassava storage roots (rMeCBM) was simulated to fit the specific root growth of KU50 (so called rMeCBM-KU50 model) and HN (so called rMeCBM-HN model) based on measurements that were taken in field conditions. Specificity of the model to cultivar was verified by simulation of root growth rate for CMC9 cassava cultivar grown under similar condition^32^. Regarding the simulation of metabolic fluxes, the results showed that half of all reactions in the carbon metabolism were required for growth in both KU50 and HN cultivars. By comparing carbon flux partitioning based on flux-sum analysis^33,34^, differences in the metabolic phenotypes of KU50 and HN including (1) carbon flux channeling towards carbohydrate biomass production, (2) carbon substrates supplied for biomass biosynthesis in plastid, and (3) metabolic balance for biomass production via the pentose phosphate pathway and the non-cyclic TCA were identified. These variations in metabolic states likely explain the difference in yield between KU50 and HN cultivars. The proposed genome-scale metabolic model of carbon assimilation is first of its kind. It is expected to be a gateway to assess the metabolism of cassava storage roots and unravel the underlying metabolic complexity of starch synthesis and accumulation in starchy roots.

## Results

### Storage root growth rate of KU50 and HN

To investigate the growth and development of cassava storage roots, KU50 and HN were propagated from stem cuttings in the field under a rainfed condition. Growth was assessed by measuring biomass accumulation in whole plant, shoot, and root during the nine-months period of study. Cultivar specific differences in whole plant and storage root growth patterns were found (Supplementary Fig. S1B–E). While HN showed gradually and near-linear increases in whole plant and storage root growth, KU50 exhibited a holding pattern as observed between 60 and 180 days after planting (DAP), before rapidly increasing (Supplementary Fig. S1B,D,E). These differences in biomass accumulation could be related to cultivar specific variations in carbon partitioning, response to environmental stressors, and nutrient reserve availability in stem cuttings used. Shoot and root growth depends on reserves of the stem cutting during the early stages of growth, before the plant becomes photosynthetically active—approximately at 30 DAP depending on cultivar^35^.

**Figure 1:**
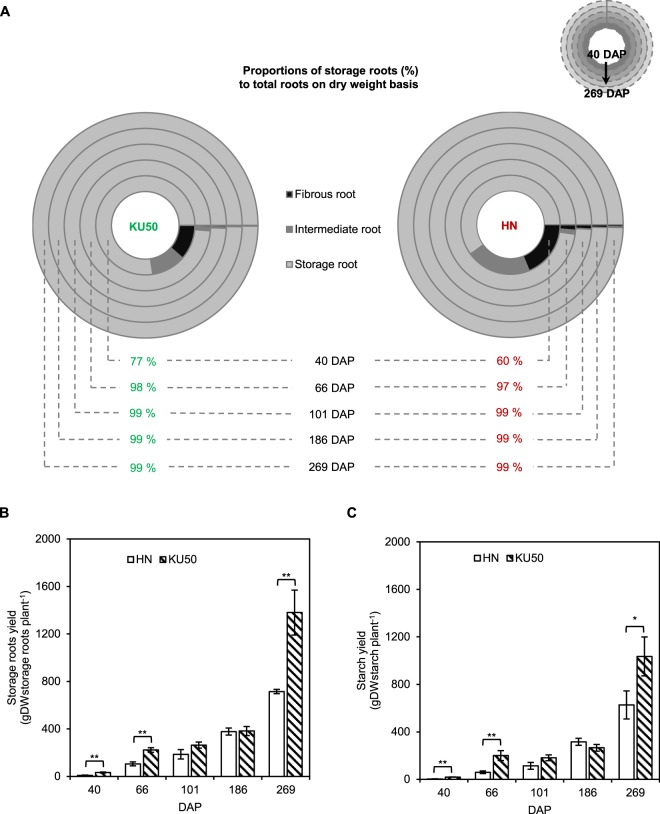
(**A**) The proportion of cassava root type (n = 4), (**B**) storage roots yield, and (**C**) root starch content, on dry weight basis, at various developmental stages of KU50 and HN cassava cultivars. Data are shown as means ± SE (n = 3). Statistical significance, based on one-sided student’s t-test, is denoted by *(*p* ≤ 0.10) or **(*p ≤ *0.05). DAP, days after planting; DW, dry weight.

At 40 DAP, storage roots made up 77% and 60% of all adventitious roots emerging from the base of stem cutting of KU50 and HN, respectively. Nearly all the roots biomass became storage roots after three months (101 DAP) (Fig. 1A). Overall, the storage root yield of KU50 (1,380.23 ± 189.46 gDW) was higher than that of HN (715.40 ± 18.16 gDW), and the difference was highly significant (p ≤ 0.05) at harvest, i.e. at approximately 269 DAP (Fig. 1B). Similarly, the starch content of KU50 was significantly higher than in HN at harvest (p ≤ 0.10; Fig. 1C), corroborating the values that were reported^14^ for KU50 and HN. We hypothesized that the variations in storage root yield and starch content are related to differences in carbon utilization towards biomass production as inferred by distinct flux partitioning between the two cultivars. Thus, constraint-based metabolic modeling of carbon assimilation was conducted to investigate carbon flux partition in storage roots of KU50 and HN. The model simulation focused on the periods that coincided with the apparent storage root enlargement and starch accumulation, from 186–269 DAP, as highlighted in gray (Supplementary Fig. S1E).

### Model of the carbon metabolism in KU50 and HN storage roots

**Figure 2:**
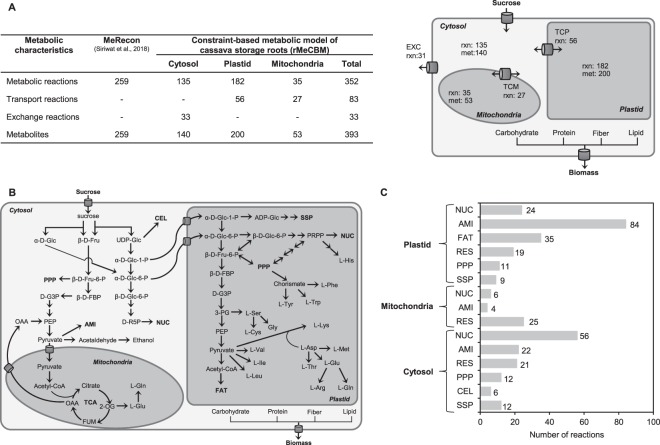
The characteristics of the constraint-based metabolic model of carbon metabolism in cassava storage roots (rMeCBM) including (**A**) summary of model components; (**B**) the carbon metabolic network; (**C**) the distribution of reactions in subcellular compartments. EXC, exchange reaction; met, number of metabolite; rxn, number of reaction; TCM, transport reaction between cytosol and mitochondria; TCP, transport reaction between cytosol and plastid. Pathway abbreviation is defined as follows: AMI, amino acid biosynthesis pathway; CEL, cell wall biosynthesis pathway; FAT, fatty acid biosynthesis pathway; NUC, nucleotide biosynthesis pathway; PPP, pentose phosphate pathway; RES, respiration pathway; SSP, starch and sucrose biosynthesis pathway. Metabolite abbreviations not defined in the text are as follows: 2-OG, 2-oxoglutarate; 3-PG, 3-phospho-D-glycerate; β-D-FBP, β-D-Fru-1,6-bisP; D-G3P, D-glyceraldehyde-3-P; D-R5P, D-ribulose-5-P; FUM, fumarate; OAA, oxaloacetate; PEP, phosphoenolpyruvate; PRPP, phosphoribosyl pyrophosphate.

The constraint-based metabolic model of cassava storage roots, rMeCBM, was constructed to simulate the intracellular carbon partitioning underlying storage root growth in KU50 and HN. The rMeCBM model covered the primary metabolism for converting sucrose (Suc) to biomass (i.e. carbohydrates, proteins, fibers, and lipid) of cassava roots. The model was basically derived from the well-defined carbon assimilation pathway of cassava, MeRecon, proposed by Siriwat^31^. MeRecon was reconstructed based on genome and biochemical information of cassava, using comparative genomics approach. It contains 259 metabolites corresponding to 259 reactions (Fig. 2A) and covers seven sub-metabolic pathways: starch and sucrose biosynthesis pathway (SSP), respiration pathway (RES), pentose phosphate pathway (PPP), cell wall biosynthesis pathway (CEL), amino acid biosynthesis pathway (AMI), fatty acid biosynthesis pathway (FAT), and nucleotide biosynthesis pathway (NUC).

MeRecon utilizes Suc as carbon substrate to produce cellular biomass of cassava storage roots (i.e. carbohydrates, proteins, fibers, and lipid). Suc, a product of photosynthesis, is transported via phloem in the shoot cells to storage roots^36^. Suc imported into root cells is either hydrolyzed by invertase (EC 3.2.1.26) to α-D-Glc and β-D-Fru or metabolized by sucrose synthase (EC 2.4.1.13) to β-D-Fru and UDP-Glc. The UDP-Glc is then used to synthesize fibers, in the form of cellulose(n + 1) and 1,4-beta-D-xylan(n + 1), in the CEL pathway. Additionally, the UDP-Glc is converted to α-D-Glc-1-P, a 6-carbon compound, which is subsequently converted to D-ribulose-5-P, a 5-carbon compound, utilized for the biosynthesis of purine in the NUC pathway. Moreover, α-D-Glc-1-P is also utilized to synthesize starch in the SSP pathway. In the PPP pathway, β-D-Fru is converted to β-D-Fru-6-P from which pyruvate, a 3-carbon compound, is synthesized in the RES pathway. In the AMI pathway, the amino acids: L-Ala, L-Arg, L-Asp, L-Asn, L-Cys, L-Glu, L-Gln, Gly, L-Ile, L-Leu, L-Lys, L-Met, L-Pro, L-Ser, L-Thr, and L-Val are synthesized from pyruvate; whereas L-His, L-Phe, L-Trp, and L-Tyr are synthesized from a 5-carbon compound. Moreover, pyruvate is utilized in the tricarboxylic acid (TCA) cycle and is also a precursor of acetyl-CoA which is utilized to synthesize hexadecanoic acid, in the FAT pathway (Fig. 2B).

To represent the complexity of eukaryotic cells, the reconstructed carbon assimilation metabolism in the cassava storage roots, rMeCBM, was partitioned into subcellular compartments: cytosol, plastid, and mitochondria based on existing data in the SWISS-PROT database^37^ and the prediction from web-based tools^38–43^. Additionally, the transport and exchange reactions that are involved in the translocation of metabolites across compartments were added to the model based upon their occurrence in other published metabolic models of Arabidopsis^44^, rice^27^, maize^45^, barley^20^, and rapeseed^46^. Subsequently, the compartmentalized model was curated by refining the atomic mass balance of each reaction and enhancing the network connectivity. Gap metabolites, which are the missing reactions in the metabolic model construction, were identified and manually filled using the data obtained from biochemical databases and literature. Following curation, the distribution of reactions in each subcellular compartment was examined (Fig. 2C). In summary, the resulting rMeCBM model composed of 468 reactions related to 393 metabolites (Fig. 2A). It included 352 biochemical reactions for metabolism, 83 transport reactions for translocation of metabolites across compartments, and 33 exchange reactions for extracellular import or export of metabolites.

To simulate the growth of KU50 and HN storage roots, cultivar specific data on sucrose uptake rate and biomass synthesis equation were input in the rMeCBM model, which hereafter was referred to as rMeCBM-KU50 and the rMeCBM-HN (details are provided in Supplementary Data S1–S4). The carbohydrates, proteins, fibers, and lipid compositions of KU50 and HN storage roots, at nine-month-old, were derived from Boonseng *et al*.^47^, and the amino acid content was obtained from Montagnac *et al*.^48^. The biomass compositions of both cassava cultivars computed from Boonseng *et al*.^47^ are shown in Supplementary Table S1. Storage roots of KU50 and HN are composed of approximately 90% carbohydrates, on dry weight basis. For KU50, the carbohydrates of storage roots consist of 84.061% starch, 6.770% Suc, 0.677% Glc, and 0.564% Fru; whereas for HN, 87.998% starch, 3.396% Suc, 0.200% Glc, and 0.499% Fru make up the carbohydrates. The mass fractions of the soluble sugars and starch were then calculated, based on 100 gDW carbohydrates (Supplementary Table S1).

The rMeCBM-KU50 and rMeCBM-HN were employed to simulate the storage root growth rate at Suc uptake rates of 0.0548 and 0.0680 mmol_Suc_ gDW^−1^_storage roots_ day^−1^ for KU50 and HN, respectively. The Suc uptake rates were calculated using measured data on leaf area and storage root dry weight as well as data on photosynthetic rate^29^ obtained from literature. Results showed that rMeCBM-KU50 and rMeCBM-HN models well imitated the growth of storage roots of both cultivars (Table 1). The optimal growth-associated ATP maintenance (*S*_*GAM*_) of the rMeCBM model was 9.8 mmol_ATP_ gDW^−1^_storage roots_ for KU50 and 14.7 mmol_ATP_ gDW^−1^_storage roots_ for HN. These values lie within the 5–42 mmol_ATP_ gDW^−1^ range reported in literature of plant metabolic models (i.e. Arabidopsis^44^, rice^27^, maize^45^, barley^20^, and rapeseed^46^). The *S*_*GAM*_ of rMeCBM-KU50 was lower than that of rMeCBM-HN, probably denoting inferior utilization of carbon substrate to produce biomass in HN relative to KU50. To assure that the model solutions were in optimal range, rMeCBM models underwent flux variability analysis (FVA) for each reaction in three compartments (cytosol, mitochondria, and plastid), and exchange and transport reactions as summarized in Supplementary Figs S1–S5, respectively.

**Table 1:** Constraints and summary of the *in silico* modeling of cassava storage roots.

Model condition	Sucrose uptake rate^*^	Maximal specific growth rate^**^	
		Experiment	Prediction
rMeCBM-KU50	0.0548	0.0090	0.0090 (*S*_GAM_^***^ = 9.8)
rMeCBM-HN	0.0680	0.0081	0.0081 (*S*_GAM_^***^ = 14.7)

^*^unit of sucrose uptake rate as mmol_Suc_ gDW^−1^_storage roots_ day^−1^.

**unit of maximal specific growth rate as day^−1^.

***unit of *S*_*GAM*_ as mmol_ATP_ gDW^−1^_storage roots_.

Regarding the simulation of rMeCBM-KU50 and rMeCBM-HN models, the sensitivity of biomass production to changes in metabolites was analyzed by shadow prices^49^. The shadow prices indicate how much does growth rate increase as a given metabolite is increasing. The results of shadow prices show that biomass biosynthesis in both cultivars was limited by similar set of metabolites, with the exception of cytosolic 6-phospho-D-gluconate which was found to activate biomass only in KU50 (Supplementary Fig. S6).

The sensitivity of biomass production to changes in reactions was analyzed by reduced costs^49^ that indicate how much does growth rate affected by each reaction flux. The reduced costs analysis (Supplementary Fig. S7) revealed the reactions that regulate storage root growth in KU50 and HN; dissimilarity in the reactions was found only in cytosol. While R01528 occurred in KU50, R02035 was instead present in HN. In the oxidative PPP, the R01528 and R02035 reactions are involved in energy production, for which D-ribulose-5-P is needed. In KU50, cytosolic 6-phospho-D-gluconate is utilized to produce D-ribulose-5-P, whereas in HN, D-6-phospho-D-gluconate is first, synthesized from glucono-1,5-lactone-6-P. Furthermore, R00235 occurred only in KU50 in the RES and R00669 was found only in HN in the AMI. Acetate is utilized by KU50 to produce acetyl-CoA in the RES and HN utilizes N-acetylornithine to produce acetate and L-Orn in the AMI.

In addition, the sensitivity of *S*_*GAM*_ required for simulating the growth rate of KU50 and HN roots was assessed by extending the band-width of the coefficient value by 20–30% (both decreasing and increasing), which allowed deviations from predicted optimal values for KU50 and HN by ±20%. The sensitivity results for both rMeCBM-KU50 and rMeCBM-HN were comparable, and the coefficient values were 7.84–12.74 mmol_ATP_ gDW^−1^_storage roots_ and 11.76–19.11 mmol_ATP_ gDW^−1^_storage roots_, respectively (Supplementary Fig. S8).

### Model specificity to represent the metabolism in a cassava cultivar

**Figure 3:**
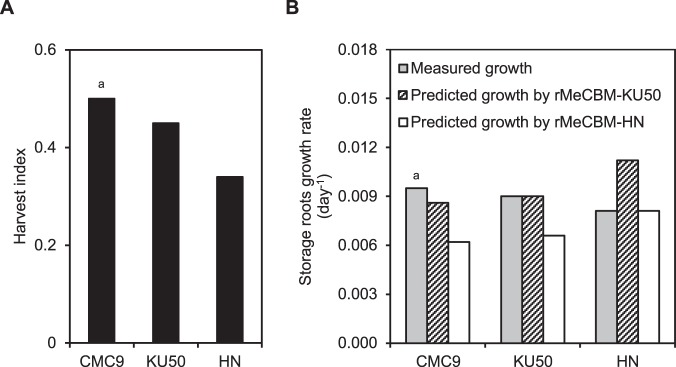
(**A**) Harvest index of CMC9, KU50, and HN cassava cultivars, on fresh weight basis. (**B**) The comparison of measured and rMeCBM-predicted storage roots growth rate of CMC9, KU50, and HN cultivars. ^a^data from Mahon *et al*.^32^.

The specificity of rMeCBM-KU50 and rMeCBM-HN to simulate the storage root growth rate of the studied cultivars was verified by employing them to simulate the storage root growth rate of CMC9 cultivar, using physiological data from Mahon *et al*.^32^. As shown in Fig. 3A, the harvest index (HI) of 0.50 reported for CMC9 is more comparable with that of KU50 (HI = 0.45) than HN (HI = 0.34), and it is indicative of the allocation of assimilated carbon to root biomass. The estimated Suc uptake rate and storage root growth rate of CMC9 were 0.0523 mmol_Suc_ gDW^−1^_storage roots_ day^−1^ and 0.0095 day^−1^, respectively. Figure 3B showed that simulated growth rate from both models deviated from experimental data; nonetheless, rMeCBM-KU50 showed superior prediction performance than rMeCBM-HN. The results may reflect the specificity of rMeCBM-KU50 and rMeCBM-HN for the cultivars.

### Distinct carbon flux partitioning underlying storage root growth in Kasetsart 50 and Hanatee cassava cultivars

**Figure 4:**
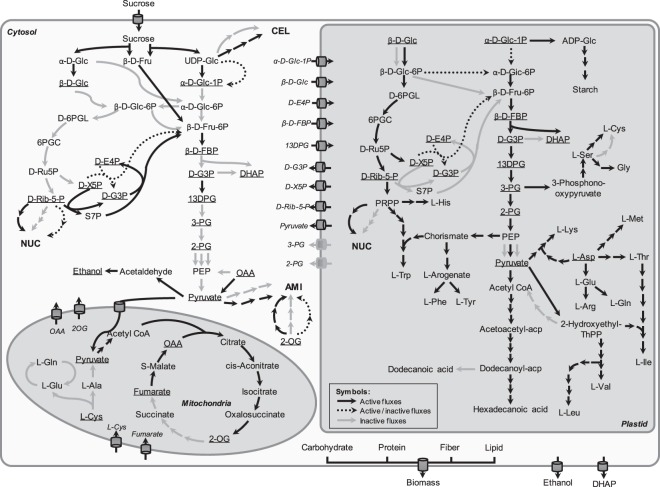
Mapping of all reaction flux distributions of rMeCBM-KU50 and rMeCBM-HN. Active fluxes, denoted by black bold lines, represent reactions containing non-zero fluxes found in both models; active/inactive fluxes, denoted by black dotted lines, represent dissimilar reactions fluxes found in both models; and inactive fluxes denoted by gray lines, represent reactions that contain zero fluxes found in both models. The underlined metabolites represent the metabolites that could transport or exchange across compartments. Metabolite abbreviations not defined in the text as follows: 13DPG, 3-phospho-D-glyceroyl-P; 2-OG, 2-oxoglutarate; 2-PG, 2-phospho-D-glycerate; 3-PG, 3-phospho-D-glycerate; 6PGC, 6-phospho-D-gluconate; β-D-FBP, β-D-Fru-1,6-bisP; D-6PGL, D-glucono-1,5-lactone-6-P; D-E4P, D-erythrose-4-p; D-G3P, D-glyceraldehyde-3-P; D-Ru5P, D-ribulose-5-P; D-X5P, D-xylulose-5-P; DHAP, glycerone-P; OAA, oxaloacetate; PEP, phosphoenolpyruvate; PRPP, phosphoribosyl pyrophosphate; S7P, sedoheptulose-7-P.

The rMeCBM model was employed to study the carbon flux partitioning underlying the distinct phenotypes of KU50 and HN cultivars. Results of carbon flux distribution analysis are provided in Fig. 4, Supplementary Figs S9,S10 and Supplementary Data S2. The flux-through reactions, i.e. active reactions denoted by black bold lines, indicate similar metabolic paths in both cultivars. Differences in the metabolic paths, i.e. active/inactive fluxes denoted by black dotted lines, represent the distinction between the cultivars (Fig. 4 and Table 2). The dissimilar reactions were found mainly in NUC, AMI, RES, PPP, and SSP pathways across compartments.

**Table 2:** Set of flux reactions with dissimilar flux values in rMeCBM-KU50 and rMeCBM-HN.

Reaction ID	Biochemical reaction	Pathway name	Flux of reaction	
			KU50	HN
R00289_c	diphosphate[c] + UDP-glucose[c] ->UTP[c] + alpha-D-glucose 1-phosphate[c]	SSP	✓	✗
R02739_c	alpha-D-glucose 6-phosphate[c]<=>beta-D-glucose 6-phosphate[c]	SSP	✗	✓
R01830_c	beta-D-fructose 6-phosphate[c] + D-glyceraldehyde 3-phosphate[c]<=>D-erythrose 4-phosphate[c] + D-xylulose 5-phosphate[c]	PPP	✗	✓
R01056_c	D-Rrbose 5-phosphate[c]<=>D-ribulose 5-phosphate[c]	PPP	✗	✓
R01051_c	ATP[c] + D-ribose[c]<=>ADP[c] + D-ribose 5-phosphate[c]	PPP	✓	✗
R02736_c	beta-D-glucose 6-phosphate[c] + NADP + [c] - > D-glucono-1,5-lactone 6-phosphate[c] + NADPH[c] + H + [c]	PPP	✗	✓
R01528_c	6-phospho-D-gluconate[c] + NADP + [c] - > D-ribulose 5-phosphate[c] + CO2[c] + NADPH[c] + H + [c]	PPP	✗	✓
R00235_c	ATP[c] + acetate[c] + CoA[c] - > AMP[c] + diphosphate[c] + acetyl-CoA[c]	RES	✗	✓
R00259_c	acetyl-CoA[c] + L-glutamate[c]<=>CoA[c] + N-acetyl-L-glutamate[c]	AMI	✗	✓
R02282_c	N-acetylornithine[c] + L-glutamate[c]<=>L-ornithine[c] + N-acetyl-L-glutamate[c]	AMI	✗	✓
R00332_c	ATP[c] + GMP[c] ->ADP[c] + GDP[c]	NUC	✓	✗
R00571_c	ATP[c] + UTP[c] + NH3[c]<=> ADP[c] + orthophosphate[c] + CTP[c]	NUC	✗	✓
R00512_c	ATP[c] + CMP[c]<=>ADP[c] + CDP[c]	NUC	✗	✓
R01227_c	GMP[c] + H2O[c]<=>guanosine[c] + orthophosphate[c]	NUC	✓	✗
R00662_c	UTP[c] + H2O[c]<=>UMP[c] + diphosphate[c]	NUC	✗	✓
R02739_p	alpha-D-glucose 6-phosphate[p]<=>beta-D-glucose 6-phosphate[p]	SSP	✓	✗
R08639_p	alpha-D-glucose 1-phosphate[p]<=>alpha-D-glucose 6-phosphate[p]	SSP	✗	✓
R01830_p	beta-D-fructose 6-phosphate[p] + D-glyceraldehyde 3-phosphate[p]<=>D-erythrose 4-phosphate[p] + D-xylulose 5-phosphate[p]	PPP	✓	✗
TCP50	alpha-D-glucose 6-phosphate[c]<=>alpha-D-glucose 6-phosphate[p]	TCP	✗	✓

✗ inactive reactions;

✓ active reactions.

**Figure 5:**
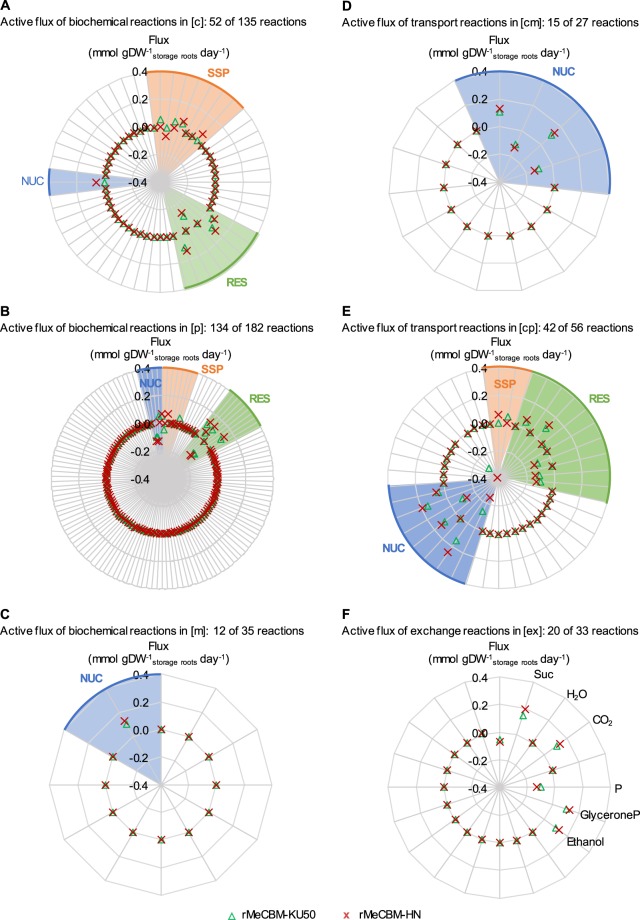
Set of active reactions distributed in (**A**) cytosol (c), (**B**) plastid (p), (**C**) mitochondria (m), (**D**) transport reactions between cytosol and mitochondria (cm), (**E**) transport reactions between cytosol and plastid (cp), and (**F**) exchange reactions (ex), in the rMeCBM-KU50 and rMeCBM-HN model. Each radar graph, the radial axes is the flux value (mmol gDW^−1^_storage roots_ day^−1^). Each line is an active reaction flux, and highlighted regions correspond to the predominant reaction fluxes in pathways. The optimal flux values were represented in green triangle for the rMeCBM-KU50 model and in red cross for the rMeCBM-HN model. Pathway abbreviation is defined as follows: AMI, amino acid biosynthesis pathway; CEL, cell wall biosynthesis pathway; FAT, fatty acid biosynthesis pathway; NUC, nucleotide biosynthesis pathway; PPP, pentose phosphate pathway; RES, respiration pathway; SSP, starch and sucrose biosynthesis pathway.

**Figure 6:**
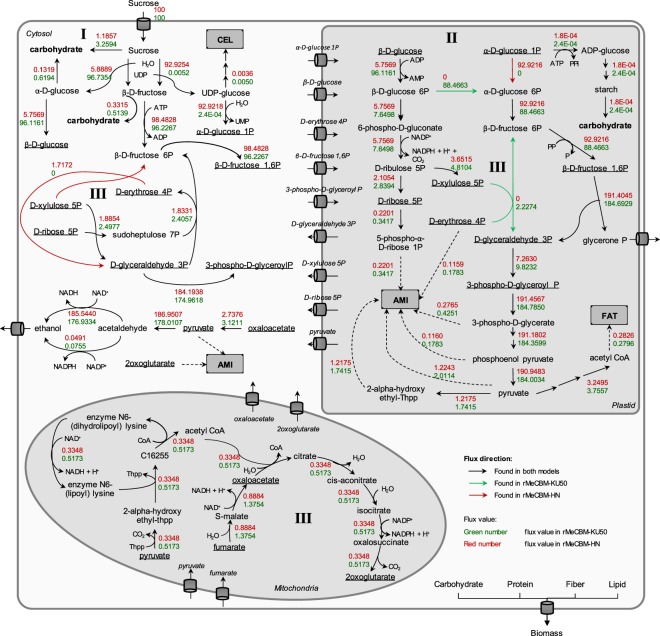
The flux sum analysis of the carbon flux distribution in the rMeCBM models of KU50 and HN. Black arrows represent flux reactions in both models; green arrows and numbers represent flux reactions and flux sum values in rMeCBM-KU50; red arrows and numbers represent flux reactions and flux sum values in rMeCBM-HN; and underlined metabolites are transport metabolites. The differences in carbon flux partitioning in both models include carbon flux channeling to carbohydrate biomass (I), carbon supplied for biomass biosynthesis in plastid (II), and metabolic balance of energy and redox in PPP and non-cyclic TCA (III).

Fluxes of the active reactions of rMeCBM-KU50 and rMeCBM-HN were compared as shown in Fig. 5. It was found that magnitude and direction of flux through the active reactions were similar in both models, and the predominant reaction fluxes were involved in SSP, RES, and NUC pathways. However, carbon flux partitioning, as determined by flux sum analysis, between KU50 and HN were not identical (Fig. 6). The two cassava cultivars prefer different paths of utilization of carbon substrate, which could reflect on their phenotypic characteristics, e.g. storage root yield, as explained below.

#### Carbon flux channeling towards storage root production

In sink organs, carbohydrates are used as the main carbon source for plant growth and development. The storage root of cassava is composed of 92% carbohydrates, on dry weight basis, which are typically stored in the form of sugars and starch (Supplementary Table S1). On the basis of 100% Suc uptake in rMeCBM, the process of carbohydrate synthesis started with direct storage of Suc in the biomass. Then, part of the Suc was converted by either invertase (EC 3.2.1.26) to α-D-Glc and β-D-Fru or sucrose synthase (EC 2.4.1.13) to β-D-Fru and UDP-Glc. Both cytosolic α-D-Glc and cytosolic β-D-Fru were also utilized for carbohydrate synthesis. Additionally, starch was synthesized in the plastid via the plastidial ADP-Glc. The simulation results revealed that KU50 and HN have different capacity to accumulate soluble sugars and starch for producing carbohydrates in storage roots. More fluxes of these compounds were distributed to carbohydrate producing reactions in KU50 than HN cassava cultivar (denoted as I in Fig. 6). Besides, KU50 tends to metabolize Suc by invertase, whereas HN mainly utilizes by sucrose synthase. Invertase cleavages the glycosidic bond and hydrolyzes sucrose into α-D-Glc and β-D-Fru. Sucrose synthase is a glycosyl transferase converting sucrose to UDP-Glc and β-D-Fru when UDP is available^36,50^. The results indicate the distinct biochemical pathway by which the two cultivars employed for sucrose utilization. Study in carrot tap roots reports evidence regarding the association of both enzymes to sugars as well as starch contents in sink organs. Repression of either enzyme results in significant reduction of soluble sugars and starch contents, whilst low activity of cell wall invertase, moreover, makes no tap root initiated in transgenic plants. The literature suggests that sucrose synthase is only relevant to growth rather than sucrose partitioning as in case of cell wall invertase^51^. The reported evidence in carrot root tap may be an explanation of sucrose utilization in KU50 and HN underlying their growth patterns (KU50—holding pattern, flipping between root-shoot; HN—gradually and near-linear).

#### (Carbon (precursor) supplied for biomass biosynthesis in plastid

The metabolic precursors of biomass including starch, amino acids, and fatty acid were imported from cytosol into plastid. Starch and fatty acid are synthesized in plastid, which is also a primary site for the production of amino acids. Moreover, the plastid is an important organelle for energy production in plants via the RES and the oxidative PPP pathways^52^. Results of the simulations revealed differences in the carbon precursors imported from cytosol and utilized in plastid (denoted as II in Fig. 6). Whereas rMeCBM-KU50 preferred importing hexose in the form of β-D-Glc, the rMeCBM-HN mostly imported hexose phosphate in the form of α-D-Glc-1-P into the plastid, where both metabolites were utilized in SSP. Additionally, in rMeCBM-KU50 model, the α-D-Glc-1-P in plastid was directly used to synthesize starch, whereas in rMeCBM-HN, it was mainly utilized to produce α-D-Glc-6-P for RES pathway and biosynthesis other biomass components.

#### Metabolic balance of energy and redox for biomass production

The simulation results indicate that the metabolic process in the mitochondrial TCA cycle was non-cyclic in both cassava root models (denoted as III in Fig. 6), corroborating Poolman *et al*.^19^ who reported non-cyclic TCA in genome-scale metabolic model of Arabidopsis, under heterotrophic suspension culture. Incomplete TCA cycle might occur in the case of different energy and redox balance for biomass production^53^.

The PPP is an important pathway for metabolic balance in cellular metabolism and is involved in regulating redox homeostasis of cells and defeating oxidative stress; in addition, D-ribulose-5-P and 5-phospho-α-D-Rib-1-P, byproducts of PPP, are utilized for nucleotide and amino acids biosynthesis, respectively^54^. The PPP can occur in both cytosol and plastid and is divided into two biochemical branches including oxidative and non-oxidative PPP. The simulation results (denoted as III in Fig. 6) revealed that carbon balance in different compartments of both cassava root models was maintained via the bypass reaction of non-oxidative PPP, R01830—the conversion of D-erythrose-4-P and D-xylulose-5-P to D-glyceraldehyde-3-P and β-D-Fru-6-P by transketolase (EC 2.2.1.1). This result is in agreement with those obtained via FVA, suggesting that R01827_C (sudoheptulose-7-P[c] + D-glyceraldehyde-3-P[c] ⇔ D-erythrose-4-P[c] + beta-D-Fru-6-P[c]) is essential for HN and R01051_C (D-Rib[c] ->D-Rib-5-P[c]) is essential for KU50 (Supplementary Fig. S11). Both D-glyceraldehyde-3-P and β-D-Fru-6-P were precursor metabolites of pyruvate in the RES pathway.

## Discussion

Global food supply faces the unprecedented threat of a rapidly changing climate together with a rapid growth in global population. Therefore, there is a need to deploy innovative approaches towards enhancing crop productivity per unit area, given the constraint of land. Cassava is a starchy staple crop and an important food security crop for poor rural households, especially in sub-Sahara Africa, who rely so much on its storage roots for their daily calorie needs. Constraint-based modeling was employed to study carbon metabolism in cassava, as we hypothesized that carbon flux partitioning is related to storage root yield.

The constraint-based metabolic model of cassava roots, rMeCBM, covered the carbon metabolism involved in the conversion of Suc to biomass compounds of cassava roots, and it was compartmentalized into cytosol, plastid, and mitochondria to replicate subcellular metabolism in cassava root cells. The rMeCBM model well simulated the specific root growth of KU50 and HN, based on comparison with field measurements. Moreover, the specificity of model to cultivar was verified by simulating the root growth rate of CMC9 cassava cultivar grown under similar condition^32^. The model verification revealed that rMeCBM-KU50 could better predict storage root growth in CMC9’s than rMeCBM-HN. The result indicated that a rMeCBM model, rather than a general model, should be specifically justified to particular cassava cultivars. This came as no surprise as the harvest index of CMC9 (0.50) was closer to that of KU50 (0.45) than HN (0.34). Model specificity was critical to identifying flux patterns responsible for the storage root yield differences in KU50 and HN cultivars. Variations in flux patterns were observed in both cultivars: during Suc import to storage root cells in cytosol, more Suc was channeled into carbohydrate synthesis in KU50 than HN; KU50 utilizes β-D-Glc as metabolic precursor of biomass production in the plastid, whereas HN utilizes α-D-Glc-1-P. KU50 uses the bypass reaction in PPP to produce energy in plastid, while HN uses the same bypass reaction to produce energy, but in cytosol.

Generally, carbon is required by cells to fuel metabolic processes and for respiration, and it is exchanged between source (mature leaves) and sink tissues (roots, young leaves, tubers, etc.) as simple sugars, primarily Suc^55^. The modeling revealed that more carbon flux was channeled towards the syntheses of carbohydrates, amino acids, and fibers in KU50 than in HN, which explains the yield gap between both cultivars. Soluble sugars and starch play a central role in carbon metabolism and may have a significant influence on the rate of plant biomass accumulation^56^. Besides, results also showed respiration was higher in HN than KU50. Maintenance respiration can represent a significant carbon cost to plants^57^. Carbon lost to respiration, as sink, further constitutes resource drawdown, which may also explain the lower storage root yield in HN.

The plastid is an important compartment for the synthesis and storage of many compounds such as starch, amino acids, fatty acids, and pigments; and these metabolic activities affect plant growth and development^58^. Based on the integration of transcriptomics data into the carbon metabolic network of cassava, α-D-Glc-1-P is mainly used as carbon source for starch biosynthesis, rather than in RES pathway, during root development of TMS60444 cassava cultivar^59^. Likewise, in KU50, α-D-Glc-1-P imported from cytosol into plastid was directly utilized for the synthesis of starch in SSP. The phenomenon appeared to be different in HN, which a majority of imported α-D-Glc-1-P was utilized for energy production in RES and to a limited extent for starch synthesis in SSP. Therefore, the cultivar-based differences in metabolite utilization reflect underlying variations in carbon flux partitioning.

Interestingly, KU50 and HN selectively utilized different precursor metabolites to generate α-D-Glc-6-P, which is able to support fatty acid biosynthesis and uses as the intermediate metabolite in PPP. Whereas α-D-Glc-6-P produced in the plastid of KU50 was derived through glucose-6-phosphate isomerase (EC 5.3.1.9) from β-D-Glc-6-P imported, that in HN was generated from α-D-Glc-1-P via phosphoglucomutase (EC 5.4.2.2). Activity of both enzymes has been proven necessitated to retain a normal starch biosynthesis in many plant species. Fernie *et al*.^60^ reported that the plastidial phosphoglucomutase activity plays a role in the control of starch and sucrose accumualtion in potato tubers. The reduced activity of plastidial phosphoglucomutase is correlated with decreased starch content, whereas sucrose content increases. Similarly, Yu *et al*.^61^ found that plastidial glucose-6-phosphate isomerase activity is correlated with starch content in leaves of Arabidopsis mutant. Glucose-6-phosphate isomerase is likely an enzyme coupling gluconeogenesis and glycolysis pathways that would help adjust the balance of anabolic and catabolic processes in cells. However, various studies demonstrate a close relationship of glucose-6-phosphate isomerase and phosphoglucomutase with other enzymes in RES and oxidative PPP in regulating metabolism in response to energy and carbon skeletons requirement of cells^62^. Up to the current knowledge, rationale by which the organisms selectively use such enzymes in sucrose metabolism is yet not fully unraveled.

## Conclusions

Overall, crop performance depends so much on source-sink relations with respect to photosynthetic carbon fixation and partitioning. Efficient nutrient uptake and mobilization for growth as well as the ability to cope with external stressors are critical. This research focused on linking carbon partitioning in cassava to storage root yield with a view to understanding how variations in flux patterns affect biomass synthesis. The constraint-based modeling revealed that carbon flux invested in the syntheses of carbohydrates and amino acids was higher in KU50 than HN, and both cultivars utilized different metabolic precursors to produce energy in plastid. The carbon flux utilized for respiration was higher in HN than in KU50. Moreover, KU50 had higher energy use efficiency; it utilized less energy to synthesize one gram of dry storage root. These results possibly explain the predominant storage root yield of KU50 and demonstrate the robustness of the rMeCBM model. The knowledge gained might be useful for identifying engineering targets for cassava yield improvement.

## Methods

### Cassava cultivation and growth measurement

Kasetsart 50 (KU50: high starch and root yield) and Hanatee (HN: low starch and root yield) cassava cultivars were propagated from stem cuttings in the field at the Rayong Field Crops Research Center, Rayong, Thailand (12°43′N, 101°08′E, 49.80 m above sea level). Plants were grown under rainfed condition (no irrigation) from September 2013 to June 2014 and no chemical fertilizer was applied during the experiment. Sixteen plants of each cassava cultivar were randomly harvested at 40, 66, 101, 186, and 269 days after planting (DAP). At the time of harvest, leaf area per plant was estimated by photography method whereby fully expanded leaves were scanned and images were analyzed using ImageJ freeware^63^. For the growth assessment, fresh weight of biomass, partitioned into above-ground (shoot) and below-ground (roots) biomass were measured. Roots emerging at the base of stem cuttings were harvested and separated into three classes: fibrous (diameter <0.5 cm), intermediate (0.5 cm ≤ diameter ≤ 1.0 cm), and storage roots (diameter > 1.0 cm) based on the stage of development before weighing from Sojikul *et al*.^64^. The root samples were subsequently freeze-dried at −50 °C until stable weight was reached, and then the dry weight was measured. All measurements were reported as mean ± SE of 16 plants. The statistical significance analysis was performed based on student’s t-test with 95% confidence (*α* ≤ 0.05).

### Soluble sugars and starch content measurement

The freeze-dried samples of storage roots were ground to powder in liquid nitrogen; 20 mg of ground sample was used to determine the soluble sugar content (i.e. Glc, Fru, and Suc) via HPLC, and 100 mg was used for measuring soluble starch content according to Jeong’s method^65^. All values were reported as mean ± SE of three plant replicates.

### Construction of constraint-based metabolic model of carbon assimilation in cassava roots

The constraint-based metabolic model of cassava storage roots (rMeCBM) was derived from the carbon assimilation pathway modified from MeRecon^31^, which was reconstructed using comparative genomic approach based on genome and biochemical information available for cassava. The model included 468 reactions involved in the conversion of Suc, as carbon substrate, to cellular biomass of cassava roots (i.e. carbohydrates, proteins, fibers, and lipid). It covered seven pathways including starch and sucrose biosynthesis (SSP), respiration (RES), pentose phosphate (PPP), cell wall biosynthesis (CEL), amino acid biosynthesis (AMI), fatty acid biosynthesis (FAT), and nucleotide biosynthesis (NUC) pathways. Subcellular compartments were assigned to these metabolic reactions based on existing data in the SWISS-PROT database^37^ and the prediction from six web-based tools: WoLF PSORT^41^, Predotar^39^, MultiLoc2^42^, CELLO^40^, TargetP^38^, and iLoc-Plant^43^. The transport and exchange reactions that are involved in the translocation of metabolites across compartments were added to the model based upon their occurrence in other plant metabolic models, including Arabidopsis^44^, barley seed^20^, maize^45^, rapeseed^46^, and rice^27^. Finally, the subcellular metabolic model was refined to ensure the balance of mass for all reactions and to attain a gap-free metabolic network. All metabolic gaps in the model were manually filled based upon reactions in biochemical databases, literature, and published plant models. The model curation and analyses were performed using COBRA Toolbox 2.0.5^66^ in MATLAB (The MathWorks, version R2015a).

### Model simulation

Anatomy and physiology of cassava roots show that approximately 85% of total weight is comprised of parenchyma cells. It is also a cell type in which starch is mainly accumulated as well as other biomass components^35,67^. Therefore, the rMeCBM model was designed to describe a parenchyma root cell by assumedly representing the cassava root cells. The rMeCBM model was built to simulate growth of storage roots in the two cassava cultivars, KU50 and HN, and also to predict carbon flux partitioning in their respective metabolism. The biomass function of the rMeCBM model was developed based on measured biomass composition including carbohydrates, proteins, lipid, and fibers in storage roots of KU50 and HN (Supplementary Table S1)^47^. Carbohydrate biomass composed of starch, Suc, Glc, and Fru; the protein biomass comprised of 18 amino acids^48^; lipid biomass was in form of hexadecanoic acid; and fiber biomass consisted of cellulose and xylan. Formulation of biomass function is provided in the Supplementary Data S1.

Suc was used as carbon substrate and was fed to the rMeCBM model. Suc uptake rate was estimated from the photosynthetic capacity of plants based upon the following assumptions:

1. The rate of CO_2_ fixation equals the photosynthetic rate of cassava. El-Sharkawy and De Tafur^29^ found that photosynthesis rate of different field grown cassava cultivars, measured on youngest fully expanded leaves at 180 DAP using LCA-2 portable system (Analytical Development Co., Hoddesdon, England), were comparable and the differences were not statistically significant. Hence, the reported average value of 16.08 µmolCO_2_ m^−2^ s^−1^ was used for the rMeCBM model. All captured CO_2_ was assumed to be fully converted to Suc in the cellular tissues of leaves.
2. Approximately 37% of the sucrose synthesized in shoot part was assumed to be allocated to underground roots for biomass production. This percentage is based on the proportion of soluble Suc to total soluble carbohydrates in shoot and root parts on dry weight basis^68^.

Accordingly, Suc uptake rates of 0.0548 and 0.0680 mmol_Suc_ gDW^−1^_storage roots_ day^−1^ for KU50 and HN, respectively were used for the rMECBM model. The model was optimized through FBA as shown in Equation (1).

1 $$Maximize\,{v}_{biomass}$$

Subject to

$$\mathop{{v}_{j,min}\le {v}_{j}\le {v}_{j,max}}\limits^{\sum _{j=1}^{n}{S}_{ij}{v}_{j}=0}$$

where, *S*_*ij*_ is the stoichiometric coefficient of metabolite *i* in reaction *j* and *n* is the set of all reactions in model. *v*_*biomass*_ is the flux of biomass reaction and *v*_*j*_ is a flux of reaction *j* which is limited in lower bound (*v*_*j,min*_) and upper bound (*v*_*j,max*_). The FBA was performed using COBRA Toolbox 2.0.5^66^. The details of the rMeCBM model of KU50 and HN are provided in the Supplementary Data S2–S4.

### Model validation

The rMeCBM models of KU50 and HN were verified for specificity to the respective cultivars by testing the models with independent data on Suc uptake rate and storage root growth rate of CMC9 cassava cultivar^32^ that was grown in similar condition as our experiment. Mahon *et al*.^32^ showed that CMC9 is physiologically closer to KU50 than HN in terms of harvest index. The rMeCBM models of KU50 and HN were employed to model the growth of CMC9 storage roots. The specificity of the rMeCBM model to the studied cultivars was deduced from the deviation of the CMC9 growth rate simulated by the KU50 and HN models.

### Flux variability analysis

To ensure optimal flux distribution, flux variability analysis (FVA)^69^ was performed for both KU50 and HN rMeCBM models. The FVA, Equation (2) below, provides sets of possible solutions by estimating the minimum and maximum values possible for each metabolic flux at the observed metabolic state.

2 $$Maximize,\,Minimize\,{v}_{j}$$

Subject to

$$\begin{array}{c}\sum _{j=1}^{n}{S}_{ij}{v}_{j}=\,0\\ \sum _{j=1}^{n}{c}_{j}{v}_{j}={Z}_{obj}\\ {v}_{j,min}\le {v}_{j}\le {v}_{j,max}\end{array}$$

where *c*_*j*_ is a vector that specifies which flux is being optimized and *Z*_*obj*_ is the value of the objective calculated from FBA. In this study, FVA was performed using COBRA Toolbox 2.0.5^66^.

### Model sensitivity analysis

The sensitivity of biomass production to changes in metabolites and reactions in the model was analyzed by shadow prices and the reduced costs^49^, respectively. The shadow prices indicate how much the corresponding metabolite would increase or decrease biomass production. Analogous to the shadow prices, the reduced costs indicate how much the corresponding reaction would increase or decrease biomass production. Both the shadow prices and the reduced costs were performed using COBRA Toolbox 2.0.5^66^.

Moreover, the sensitivity of model to *S*_*GAM*_ was analyzed. The *S*_*GAM*_ is the energy requirement for root growth, and it is located in the biomass biosynthesis reaction (reaction no.: v351) in the form of a stoichiometric coefficient of ATP, ADP, and P. Changes in predicted growth rate, i.e. biomass flux, relative to optimal point in response to alteration of *S*_*GAM*_ were investigated in Equation (3):

3 $$\varepsilon ( \% )=\frac{{v}_{p}-{v}_{o}}{{v}_{o}}\times 100$$

where, *ε* is the percentage error of model simulation to measured cassava storage root growth rate. *v*_*p*_ and *v*_*o*_ are the storage roots growth rates predicted from perturbed and optimal conditions, respectively.

### Flux-sum analysis of carbon flux distribution

Carbon flux partitioning was calculated from the predicted metabolic flux distribution based on flux-sum approach^33,34^. Flux-sum analysis is a metabolite-centric approach which sums up all influxes or effluxes around the metabolite. It explains the phenotypic changes at each metabolite level but is unable to represent the direction and quantity of change. To overcome these limitations, flux-sum analysis was modified as demonstrated in Equation (4). Basically, the flux sum of each metabolite was calculated from surrounding flux metabolites based on the percentage of original flux-sum approach. The flux sum of the original metabolites was calculated based on Suc uptake rate of 100%.

4 $$flux\,su{m}_{{M}_{i}{R}_{j}}( \% )=\frac{{\sum }^{}{S}_{{M}_{i}{R}_{j}}\times |\,flu{x}_{{M}_{i}{R}_{j}}\,|}{{\sum }^{}{S}_{{M}_{i}{R}_{k}}\times |\,flu{x}_{{M}_{i}{R}_{k}}\,|}\times {\sum }^{}flux\,su{m}_{{M}_{i}{R}_{k}}$$

where,$\,{S}_{{M}_{i}{R}_{j}}$ is the stoichiometric number of metabolite *i* in reaction *j*, which consumes metabolite *i* to produce its derivatives. ${S}_{{M}_{i}{R}_{k}}$ is the stoichiometric number of metabolite *i* at reaction *k*, what generates metabolite *i* from its substrate metabolites. $flux{}_{{M}_{i}{R}_{j}}$ is the magnitude of flux through reaction *j*, which consumes metabol*i*te *i* to produce its derivatives. $flux{}_{{M}_{i}{R}_{k}}$ is the magnitude of flux through reaction *k*, which generates metabol*i*te *i* from its substrate metabolites.

## Supplementary information

Supplementary Dataset 2

Supplementary Dataset 3

Supplementary Dataset 4

Supplementary Information
