# Supplementary material for: Understanding carbon utilization routes between high and low starch-producing cultivars of cassava through Flux Balance Analysis
Source: Sci Rep. 2019 Feb 27;9:2964. doi: 10.1038/s41598-019-39920-w (PMC6393550; doi:10.1038/s41598-019-39920-w)
Supplement: Supplementary file 4 — Supplementary Information [file 41598_2019_39920_MOESM4_ESM.pdf]

Understanding carbon utilization routes between high and low starch-producing cultivars of cassava through Flux Balance Analysis

Porntip Chiewchankaset[1], Wanatsanan Siriwat[2], Malinee Suksangpanomrung[3], Opas Boonseng[4], Asawin Meechai[2,5], Morakot Tanticharoen[6], Saowalak Kalapanulak[2,7*], and Treenut Saithong[2,7*]

[1] Biotechnology Program, School of Bioresources and Technology, King Mongkut's University of Technology Thonburi (Bang Khun Thian), Bangkok, 10150, Thailand
[2] Systems Biology and Bioinformatics Research Group, Pilot Plant Development and Training Institute, King Mongkut's University of Technology Thonburi (Bang Khun Thian), Bangkok, 10150, Thailand
[3] Plant Molecular Genetics and Biotechnology Laboratory, National Center for Genetic Engineering and Biotechnology, Thailand Science Park, Pathumthani, 12120, Thailand
[4] Rayong Field Crops Research Center, Department of Agriculture, Rayong, 21150, Thailand
[5] Department of Chemical Engineering, Faculty of Engineering, King Mongkut's University of Technology Thonburi (Bang Mod), Bangkok, 10140, Thailand
[6] School of Bioresources and Technology, King Mongkut's University of Technology Thonburi (Bang Khun Thian), Bangkok, 10150, Thailand
[7] Bioinformatics and Systems Biology Program, School of Bioresources and Technology, King Mongkut's University of Technology Thonburi (Bang Khun Thian), Bangkok, 10150, Thailand
[*]E-mail: saowalak.kal@kmutt.ac.th and treenut.sai@kmutt.ac.th

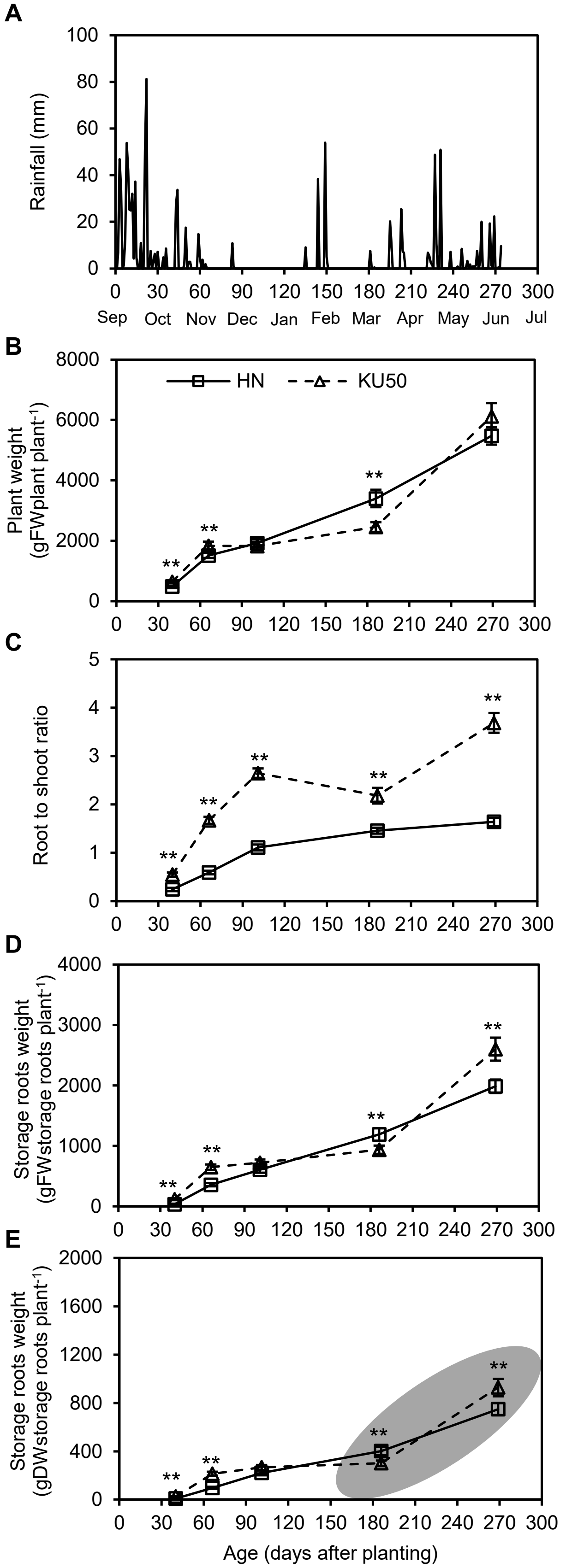

Figure S1. The growth patterns of KU50 and HN cassava cultivars related with **A)** rainfall distribution during the period of study which was collected from Thai Meteorological Department at Huai Pong station, Rayong. The growth pattern was measured based on plant biomass weight, including **B)** total plant fresh weight, **C)** root to shoot ratio, **D)** storage root fresh weight, and **E)** storage root dry weight under rainfed field condition at different growth stages. Data are shown as means±SE (n = 16). ** denotes significant difference at $p \leq 0.05$, based on one-sided student's t-test. Highlighted region in gray is the focused periods for model simulation.

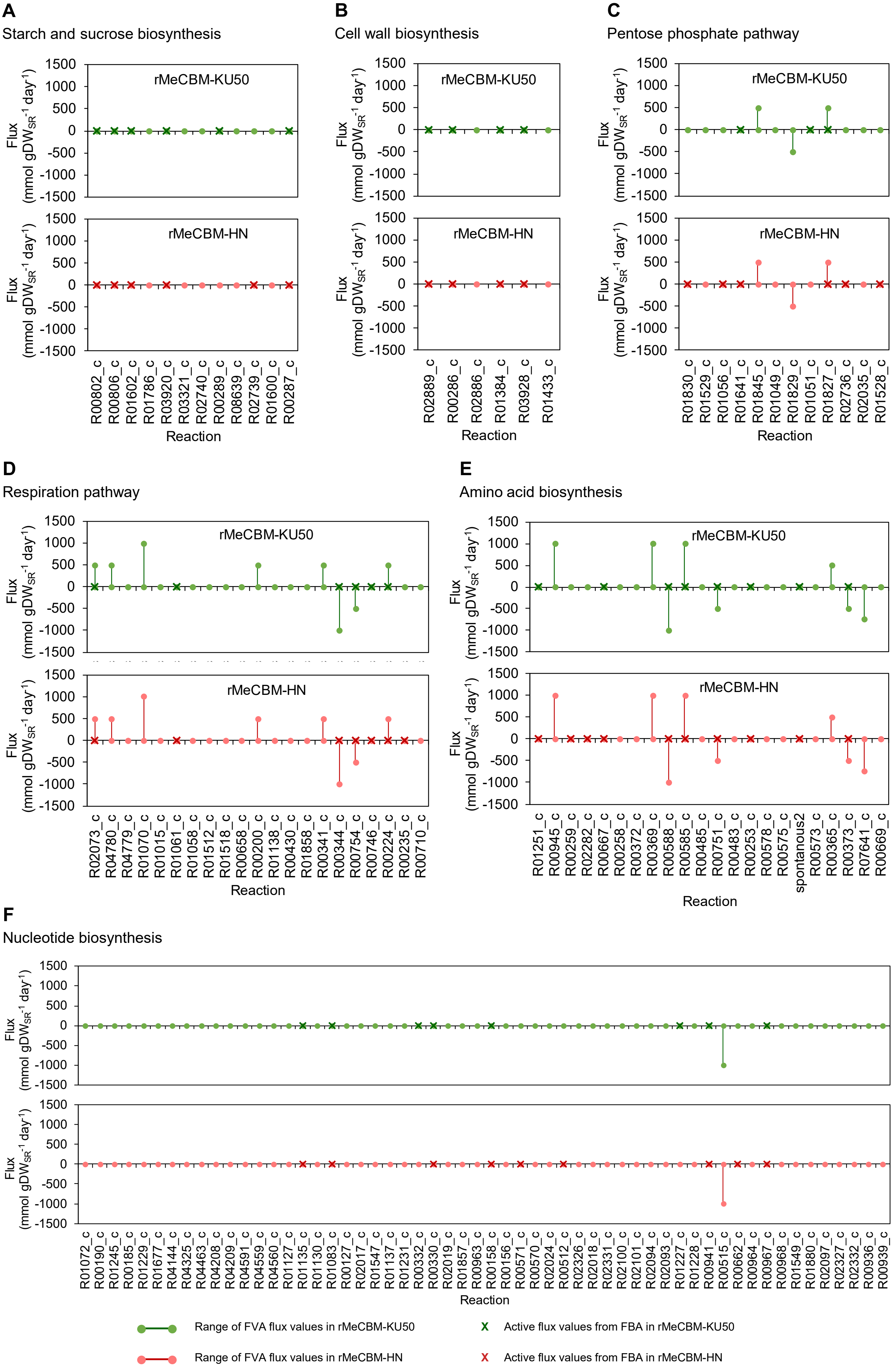

Figure S2. The minimum and maximum reaction fluxes in mmol gDW^{-1}$_{\text{storage roots}}$ day^{-1} from FVA and predicted active flux from FBA in: **A)** starch and sucrose biosynthesis, **B)** cell wall biosynthesis, **C)** pentose phosphate pathway, **D)** respiration pathway, **E)** amino acid biosynthesis, and **F)** nucleotide biosynthesis in cytosol.

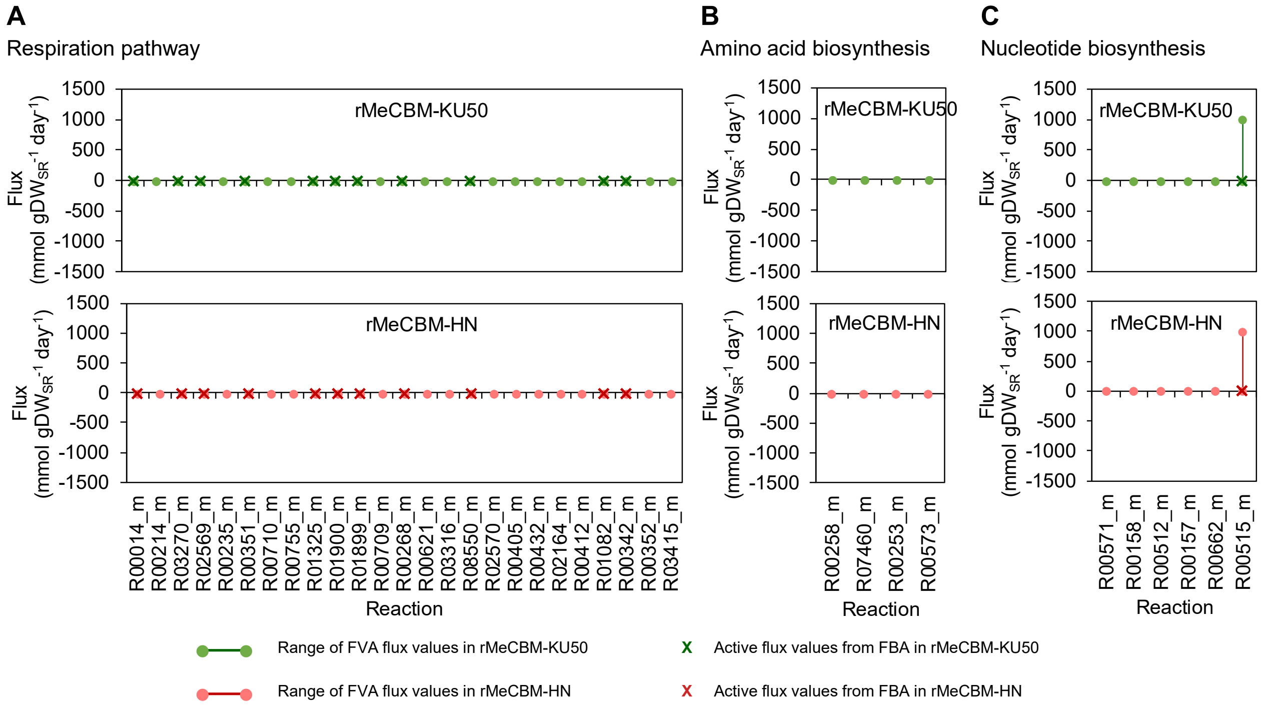

Figure S3. The minimum and maximum reaction fluxes in mmol $gDW^{-1}{}_{storage\ roots}$ day^{-1} from FVA as well as the predicted active flux from FBA in: **A)** respiration pathway, **B)** Amino acid biosynthesis, and **C)** nucleotide biosynthesis in mitochondria.

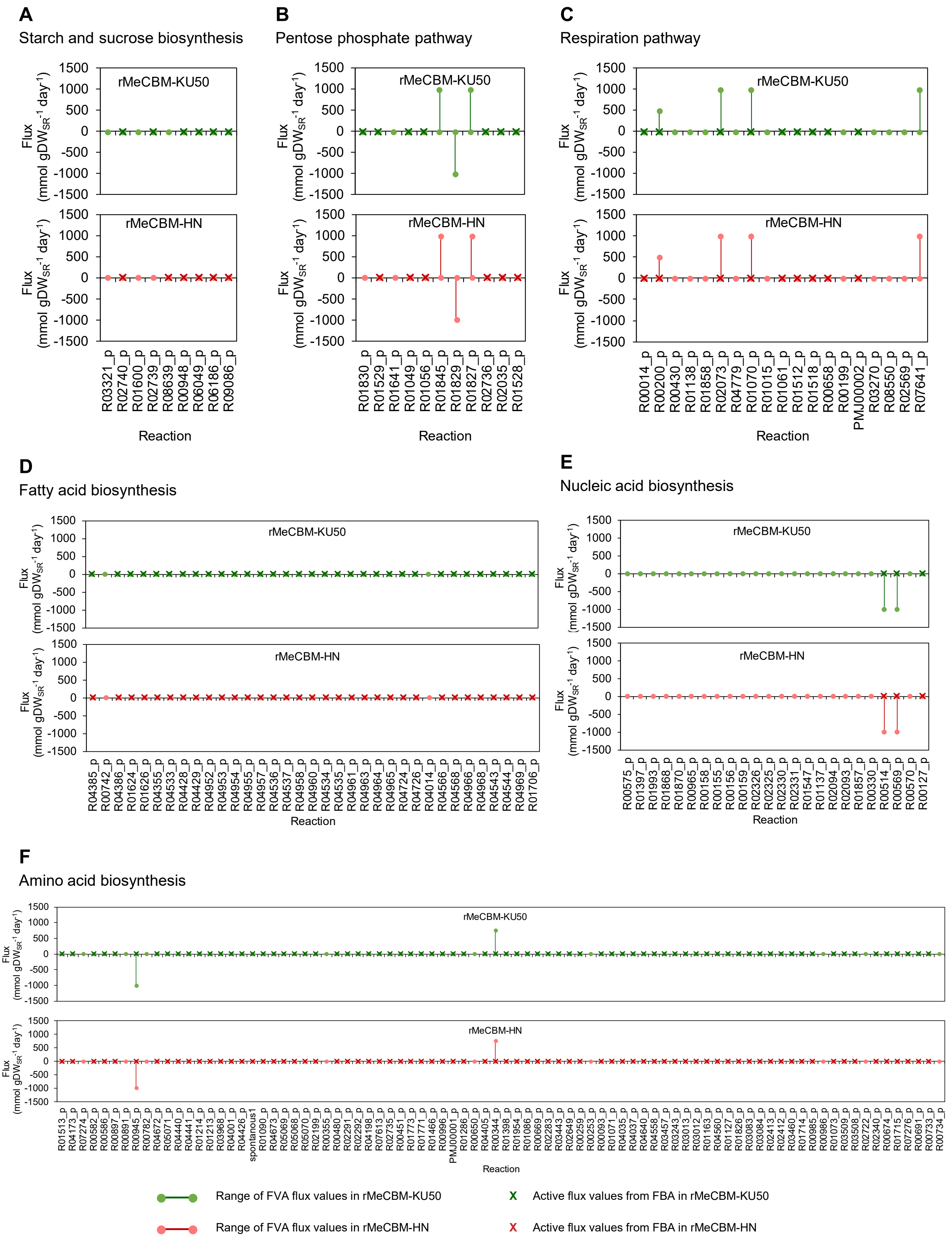

Figure S4. The minimum and maximum reaction fluxes in mmol gDW^{-1}$_{\text{storage roots}}$ day^{-1} from FVA as well as the predicted active flux from FBA in: **A)** starch and sucrose biosynthesis, **B)** pentose phosphate pathway, **C)** respiration pathway, **D)** fatty acid biosynthesis, **E)** nucleotide biosynthesis, and **F)** amino acid biosynthesis in plastid.

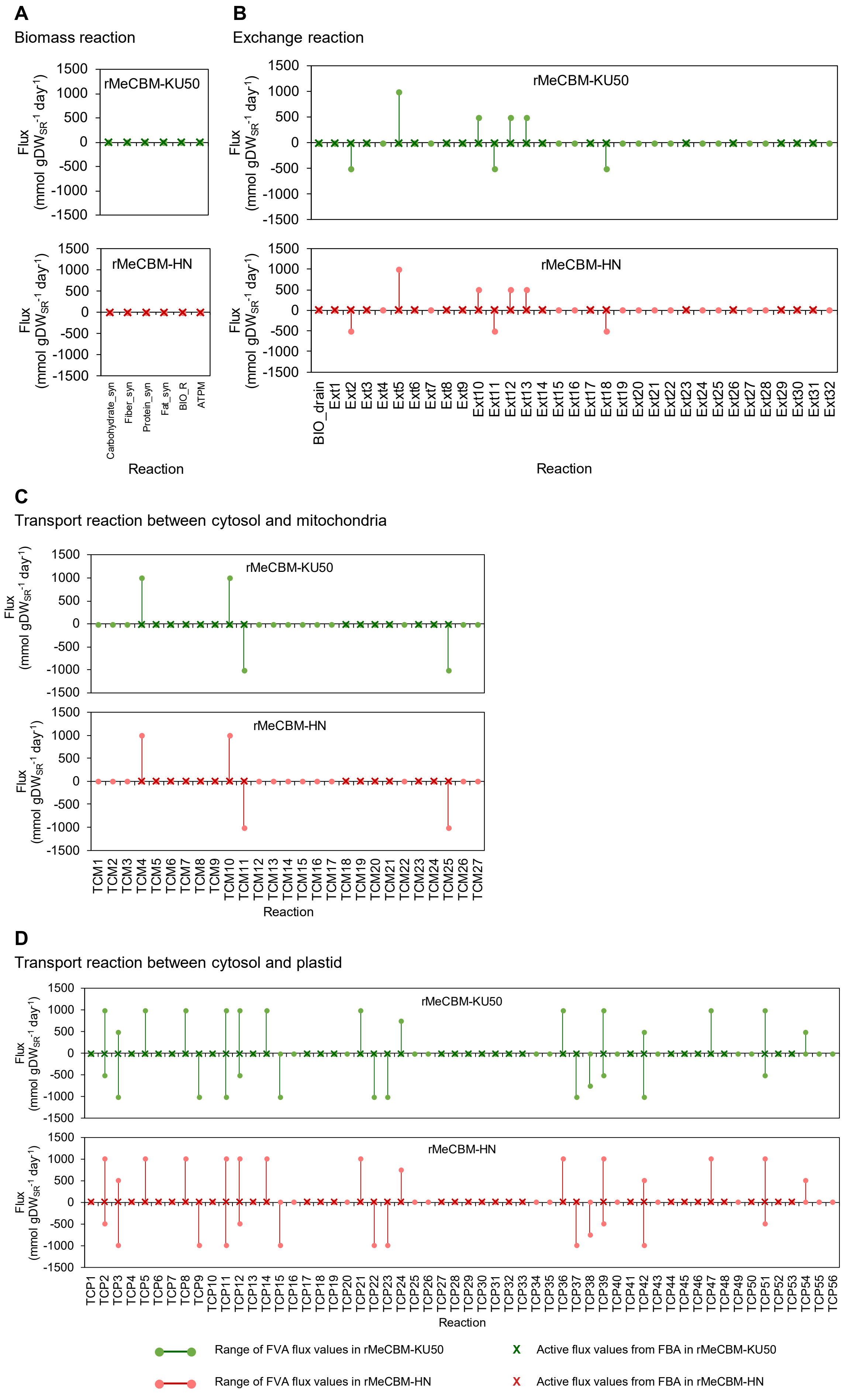

Figure S5. The minimum and maximum reaction fluxes in mmol $gDW^{-1}_{storage\ roots}$ day^{-1} from FVA as well as the predicted active flux from FBA in: A) biomass reaction, B) exchange reaction, C) transport reaction between cytosol and mitochondria, and D) transport reaction between cytosol and plastid.

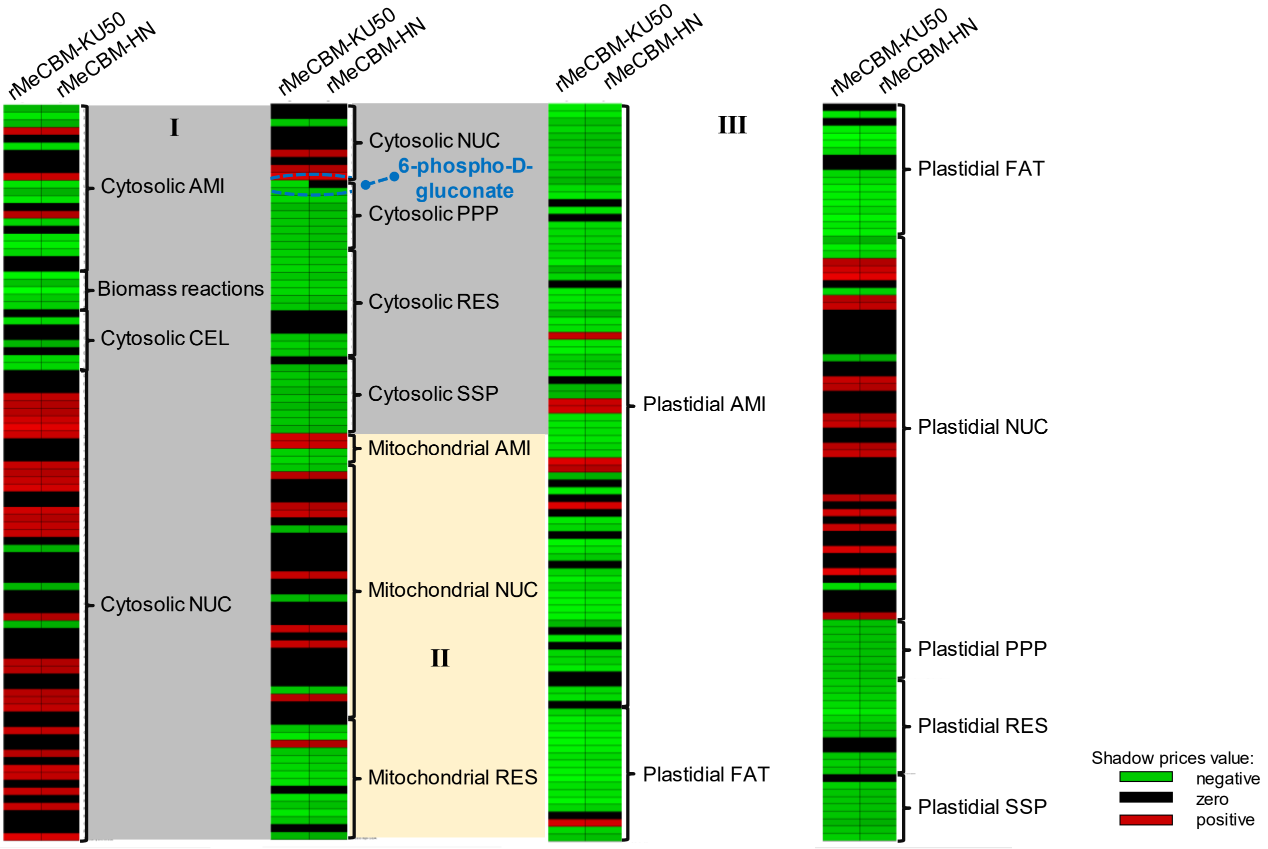

Figure S6. Shadow prices for each metabolite of rMeCBM-KU50 and rMeCBM-HN. AMI: amino acid biosynthesis pathway; CEL: cell wall biosynthesis pathway; FAT: fatty acid biosynthesis pathway; NUC: nucleotide biosynthesis pathway; PPP: pentose phosphate pathway; RES: respiration pathway; and SSP: starch and sucrose biosynthesis pathway. Set of metabolites distributed in cytosol (I), mitochondria (II), and plastid (III).

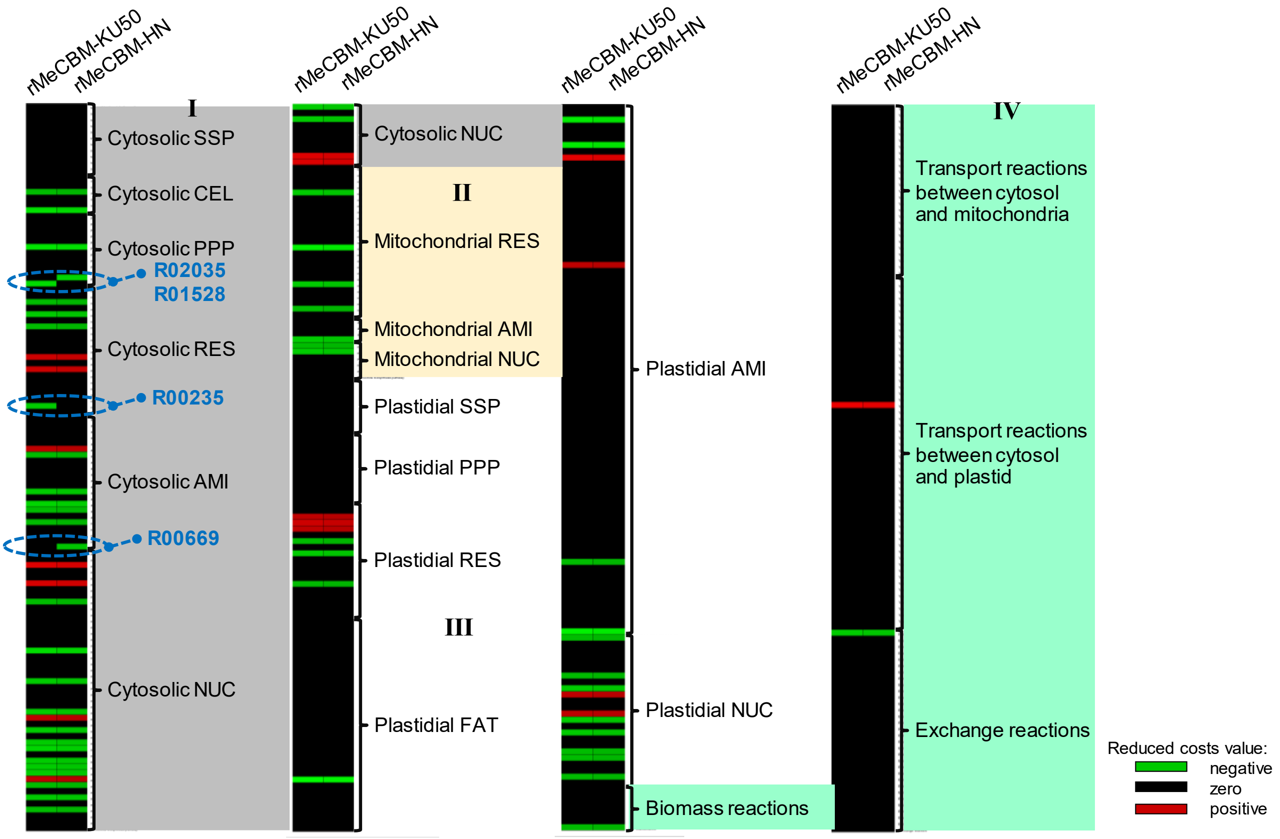

Figure S7. Reduced costs for each reaction of rMeCBM-KU50 and rMeCBM-HN. AMI: amino acid biosynthesis pathway; CEL: cell wall biosynthesis pathway; FAT: fatty acid biosynthesis pathway; NUC: nucleotide biosynthesis pathway; PPP: pentose phosphate pathway; RES: respiration pathway; and SSP: starch and sucrose biosynthesis pathway. Set of reactions distributed in cytosol (I), mitochondria (II), and plastid (III). Set of biomass, transport, and exchange reactions denoted in IV.

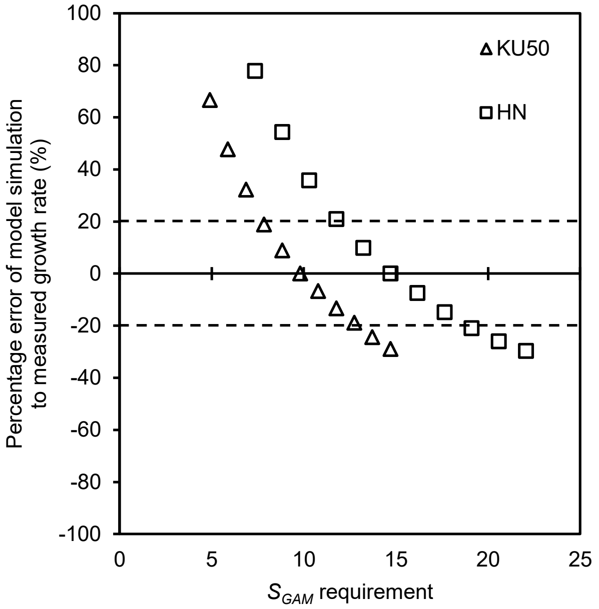

Figure S8. Sensitivity analysis of S_{GAM} to predicted growth rate of the rMeCBM models. Triangles and squares represent S_{GAM} of rMeCBM-KU50 and rMeCBM-HN, respectively. Dotted lines lay out the percentage error in the range of ±20% of model simulation to measured storage root growth rate.

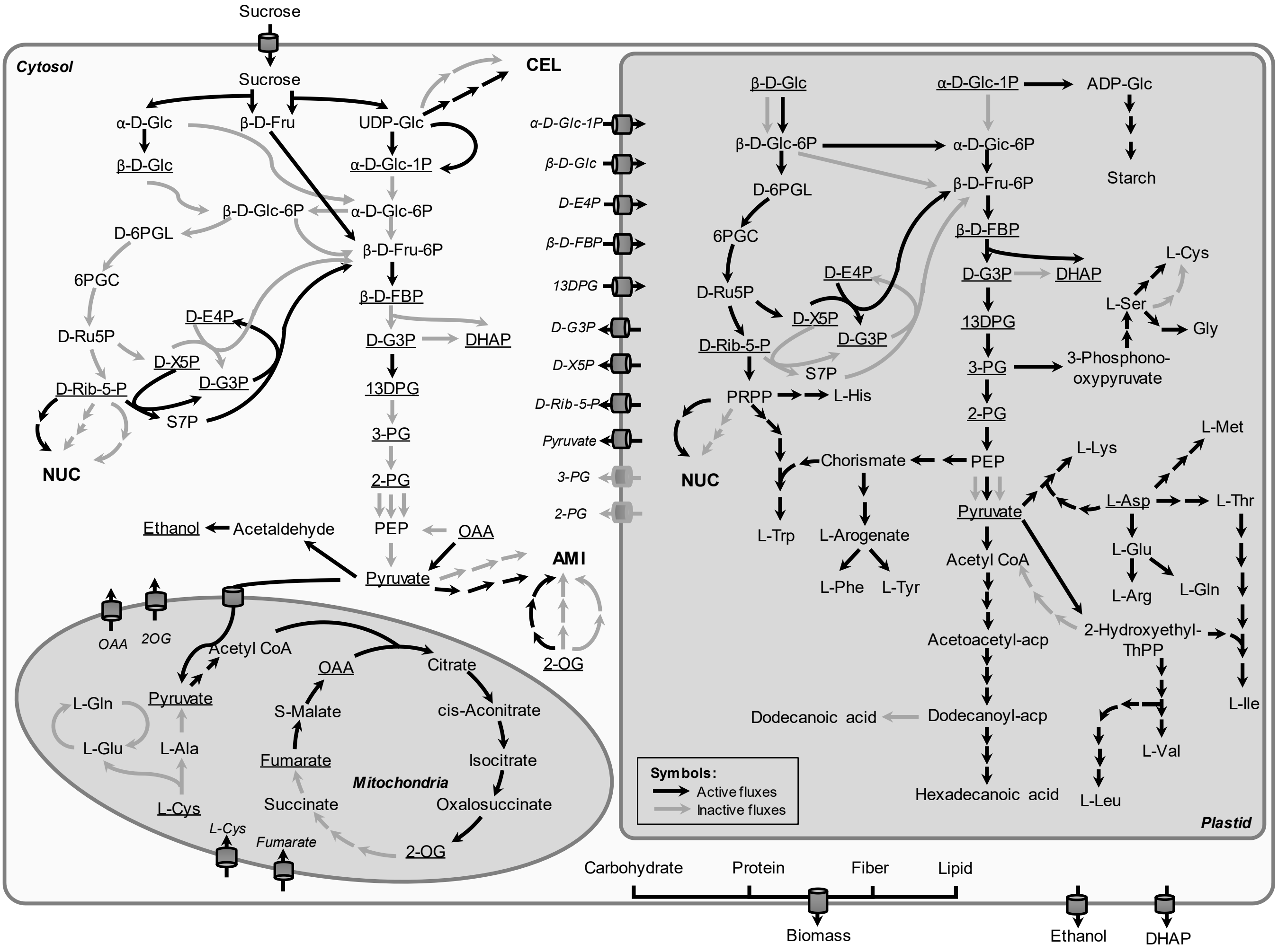

Figure S9. Mapping of all reaction flux distributions of rMeCBM-KU50 model. Active fluxes, denoted by black bold lines, represent reactions containing non-zero fluxes, and inactive fluxes denoted by gray lines, represent reactions that contain zero fluxes. The underlined metabolites represent the metabolites that could transport or exchange across compartments. Metabolite abbreviations not defined in the text as follows: 13DPG, 3-phospho-D-glyceroyl-P; 2-OG, 2-oxoglutarate; 2-PG, 2-phospho-D-glycerate; 3-PG, 3-phospho-D-glycerate; 6PGC, 6-phospho-D-gluconate; β-D-FBP, β-D-Fru-1,6-bisP; D-6PGL, D-glucono-1,5-lactone-6-P; D-E4P, D-erythrose-4-p; D-G3P, D-glyceraldehyde-3-P; D-Ru5P, D-ribulose-5-P; D-X5P, D-xylulose-5-P; DHAP, glycerone-P; OAA, oxaloacetate; PEP, phosphoenolpyruvate; PRPP, phosphoribosyl pyrophosphate; S7P, sedoheptulose-7-P.

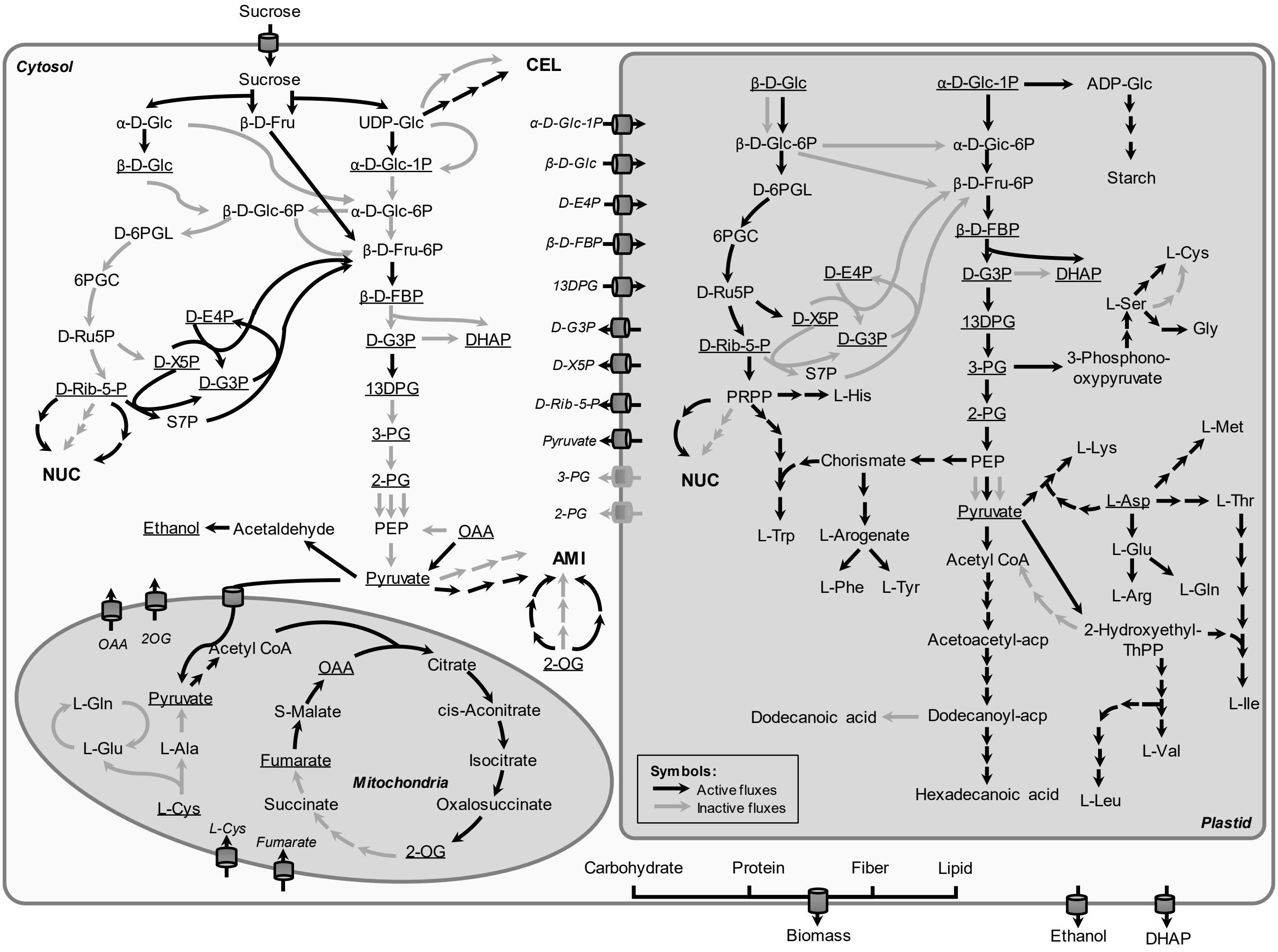

Figure S10. Mapping of all reaction flux distributions of rMeCBM-HN model. Active fluxes, denoted by black bold lines, represent reactions containing non-zero fluxes, and inactive fluxes denoted by gray lines, represent reactions that contain zero fluxes. The underlined metabolites represent the metabolites that could transport or exchange across compartments. Metabolite abbreviations not defined in the text as follows: 13DPG, 3-phospho-D-glyceroyl-P; 2-OG, 2-oxoglutarate; 2-PG, 2-phospho-D-glycerate; 3-PG, 3-phospho-D-glycerate; 6PGC, 6-phospho-D-gluconate; β-D-FBP, β-D-Fru-1,6-bisP; D-6PGL, D-glucono-1,5-lactone-6-P; D-E4P, D-erythrose-4-p; D-G3P, D-glyceraldehyde-3-P; D-Ru5P, D-ribulose-5-P; D-X5P, D-xylulose-5-P; DHAP, glycerone-P; OAA, oxaloacetate; PEP, phosphoenolpyruvate; PRPP, phosphoribosyl pyrophosphate; S7P, sedoheptulose-7-P.

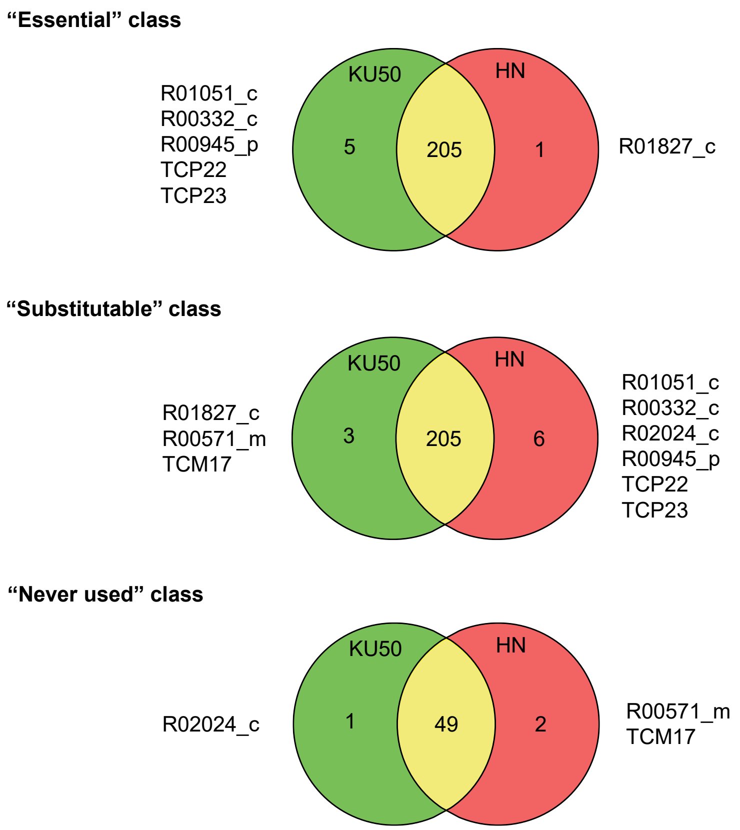

Figure S11. Set of flux reactions from FVA analysis in the rMeCBM model of KU50 and HN. Essential class is reactions that carry non-zero fluxes; Substitutable class is reactions that carry both zero and non-zero fluxes; and Never used class is reactions that carry zero fluxes.

Biomass components	KU50		HN	
	**Contents (%gDW)**	**%Mass fraction**	**Contents (%gDW)**	**%Mass fraction**
**Carbohydrate** [a]	92.072	100.000	92.093	100.000
Starch	84.061	91.299	87.998	95.553
Sucrose	6.770	7.353	3.396	3.688
Glucose	0.677	0.735	0.200	0.217
Fructose	0.564	0.613	0.499	0.542
**Protein** [a]	5.783	100.000	5.134	100.000
Alanine [b]	0.612	10.575	0.543	10.575
Arginine [b]	1.180	20.408	1.048	20.408
Aspartate [b]	0.526	9.019	0.467	9.019
Cystine [b]	0.043	0.742	0.038	0.742
Glutamate [b]	0.612	10.575	0.543	10.575
Glycine [b]	0.043	0.742	0.038	0.742
Histidine [b]	0.279	4.824	0.248	4.824
Isoleucine [b]	0.107	1.855	0.095	1.855
Leucine [b]	1.255	21.707	1.114	21.707
Lysine [b]	0.279	4.824	0.248	4.824
Methionine [b]	0.107	1.855	0.095	1.855
Phenylalanine [b]	0.107	1.855	0.095	1.855
Proline [b]	0.107	1.855	0.095	1.855
Serine [b]	0.161	2.783	0.143	2.783
Threonine [b]	0.107	1.855	0.095	1.855
Tryptophan [b]	0.054	0.928	0.048	0.928
Tyrosine [b]	0.043	0.742	0.038	0.742
Valine [b]	0.161	2.783	0.143	2.783
**Fiber** [a]	1.693	100.000	2.151	100.000
Cellulose [c]	0.846	50.000	1.075	50.000
Xylan [c]	0.846	50.000	1.075	50.000
**Lipid** [a]	0.451	100.000	0.623	100.000
Hexadecanoic acid [c]	0.451	100.000	0.623	100.000

[a] data from Boonseng et al., 1999
[b] data from Montagnac et al., 2009
[c] data from assumption

Table S1 The biomass composition of cassava storage roots at nine months after planting, based on 100 g dry weight (gDW) of storage roots.
